# A broadband, self-powered, and polarization-sensitive PdSe_2_ photodetector based on asymmetric van der Waals contacts

**DOI:** 10.1515/nanoph-2022-0660

**Published:** 2023-01-11

**Authors:** Xuran Zhang, Mingjin Dai, Wenjie Deng, Yongzhe Zhang, Qi Jie Wang

**Affiliations:** School of Electrical and Electronic Engineering, Nanyang Technological University, 50 Nanyang Avenue, Singapore 639798, Singapore; Key Laboratory of Optoelectronics Technology, Ministry of Education, Faculty of Information Technology, Beijing University of Technology, Beijing 100124, China; Key Laboratory of Optoelectronics Technology, Ministry of Education, Faculty of Information Technology, Beijing University of Technology, Beijing 100124, China

**Keywords:** asymmetric Schottky barriers, PdSe_2_, polarization sensitive, self-powered, van der Waals contacts

## Abstract

Self-powered photodetectors with broadband and polarization-sensitive photoresponse are desirable for many important applications such as wearable electronic devices and wireless communication systems. Recently, two-dimensional (2D) materials have been demonstrated as promising candidates for self-powered photodetectors owing to their advantages in light–matter interaction, transport, electronic properties, and so on. However, their performance in speed, broadband response, and multifunction is still limited. Here, we report a PdSe_2_ photodetector with asymmetric van der Waals (vdWs) contacts formed by using a homojunction configuration. This device achieves a high responsivity approaching 53 mA/W, a rise/decay time of about 0.72 ms/0.24 ms, and a detectivity of more than 5.17 × 10^11^ Jones in the visible-near infrared regime (532–1470 nm). In addition, a linear polarization-sensitive response can be observed with an anisotropy ratio of 1.11 at 532 nm and 1.62 at 1064 nm. Furthermore, a strong anisotropic response endows this photodetector with outstanding polarization imaging capabilities, realizing a contrast-enhanced degree of linear polarization imaging. Our proposed device architecture demonstrated the great potential of PdSe_2_-based asymmetric vdWs contacts for high-performance photodetectors operating without any external bias.

## 1 Introduction

Self-powered photodetectors are criteria for realizing low power consumption in integrated systems [1]. Traditional photodetectors require a high external bias to obtain a detectable photocurrent. Thus their performance in suppressing dark current, eliminating circuit noise, and low-power operation is usually limited [2–4]. Generally, several methods can be considered for constructing self-powered photodetectors: (i) utilizing photovoltaic effect in p-n junction (including heterojunction and homojunction) photodetectors [5–7]; (ii) generating photovoltaic signals in a Schottky junction [8, 9]; (iii) using a temperature gradient to drive the carriers based on photothermoelectric (PTE) effect [10–12]; (iv) designing self-powered photodetectors based on ferroelectric materials assisted with spontaneous polarization [13–15]. On the other hand, different mechanisms lead to different photoresponse speeds which are important for some practical applications like optical communication, imaging system, and high-speed optical chips [15–17]. Given the speed of a photodetector, the device based on a Schottky junction usually exhibits a faster speed than other types of phototransistors because the defect-induced minority carrier trapping can cause the devices to operate slower [18]. Various methods can be applied to enhance the self-powered and high-speed properties of a photodetector. Among them, coupling a basic device with asymmetric contacts receives much attention to their great potential in achieving self-powered photodetectors with large Schottky contact differences. It has shown that such photodetectors exhibit high performance in their electric and optoelectrical properties [19, 20].

Self-powered photodetectors based on a heterojunction structure usually require complicated fabrication processes, which may limit their further applications [21, 22]. Through forming two different contacts at the two terminals of active material, asymmetric contacts for photodetectors can be realized which exhibit outstanding properties such as low power consumption, broadband detection, high responsivity [23–26], and so on. Different metal electrodes with different work functions can construct Schottky barriers with different heights in the two terminals, thus generating a self-driven net photocurrent. In addition, the geometry of the contacts in the two terminals can also be modulated to be asymmetric, to produce a self-powered photodetector [22]. Furthermore, electrodes are not necessarily limited to metal materials, other 2D materials like graphene with extraordinary electric and transport properties are promising candidates and can also be assembled as an electrode. Self-powered photodetectors based on this structure exhibit high performance in photodetection [10]. However, the traditional methods for constructing asymmetric contacts like sputtering and evaporation are strongly affected by the Fermi level pinning effect, which makes it difficult to form an asymmetric contact with a strong photovoltaic effect in the interface [27]. Recently, van der Waals (vdWs) contacts as a new fabrication method to suppress the Fermi level pinning effect has received great interest. For 2D materials, they can form vdWs contact with metal or 2D semimetal materials through vdWs forces owing to their pristine interfaces free of dangling bonds. Recently, extensive research efforts have been devoted to investigating the unique electrical and optoelectrical properties of various 2D materials which are promising for light–matter interaction [28, 29], transport [30], and electronics [31]. Transition-metal dichalcogenides (TMDs) materials, coupled with layered crystal structure [32], strong anisotropy absorption [33, 34], and tunable bandgap [35, 36], appear to be promising candidates in fabricating advanced photodetectors. Till now, various TMDs materials have been studied to enhance the photoresponse of photodetectors, such as MoS_2_ [37, 38], WSe_2_ [22, 39], and WS_2_ [40, 41]. As an emerging candidate in TMDs materials, PdSe_2_ has strong interaction between the material layers [42], indirect energy band structure [43], and outstanding anisotropic properties [35]. Similar to other kinds of TMDs materials, the band gap of PdSe_2_ nanoflakes is apparently influenced by modulating the material thickness [44, 45]. However, an important difference between PdSe_2_ and many other kinds of 2D materials is that PdSe_2_ has a tunable bandgap ranging from 0 eV for the bulk to 1.3 eV for the monolayer, which means that the properties of PdSe_2_ can transit from semiconductor to semimetal by controlling the nanoflake thickness [46, 47]. This paves a new way to construct unique vdWs contacts between semiconductors and semimetals.

Herein, a PdSe_2_ photodetector with asymmetric vdWs contacts was successfully fabricated by using a PdSe_2_ homojunction and a bottom Au electrode. Leveraging the intrinsic interlayer vdWs force, one vdWs contact is formed at the interface between the thick and thin PdSe_2_ layers. The other vdWs contact is formed at the interface between the thin PdSe_2_ layer and the bottom Au electrode. Due to the different work functions between Au and semimetal thick PdSe_2_ flakes, asymmetric Schottky barriers at two vdWs contacts were achieved in the two terminals of the thin PdSe_2_ region. Under global illumination, this PdSe_2_ photodetector was enabled to achieve high-performance photodetection at zero bias. Significantly, a broadband detection from visible to the near-infrared regime (532 nm–1470 nm), a fast response speed with a response time of 0.72 ms/0.24 ms, a high responsivity (53 mA/W at 730 nm), and a high detectivity of over 5.17 × 10^11^ Jones under zero bias was achieved in this PdSe_2_ photodetector. Its anisotropy sensitivity was verified using lasers with different wavelengths (532 nm and 1064 nm), presenting promising polarized light detection ability, with an anisotropy ratio reaching 1.11 at 532 nm and 1.62 at 1064 nm. Especially, a polarization imaging with a contrast-enhanced degree of linear polarization (DoLP) was demonstrated by this device, showing excellent polarization imaging capabilities.

## 2 Result and discussion

**Figure 1:**
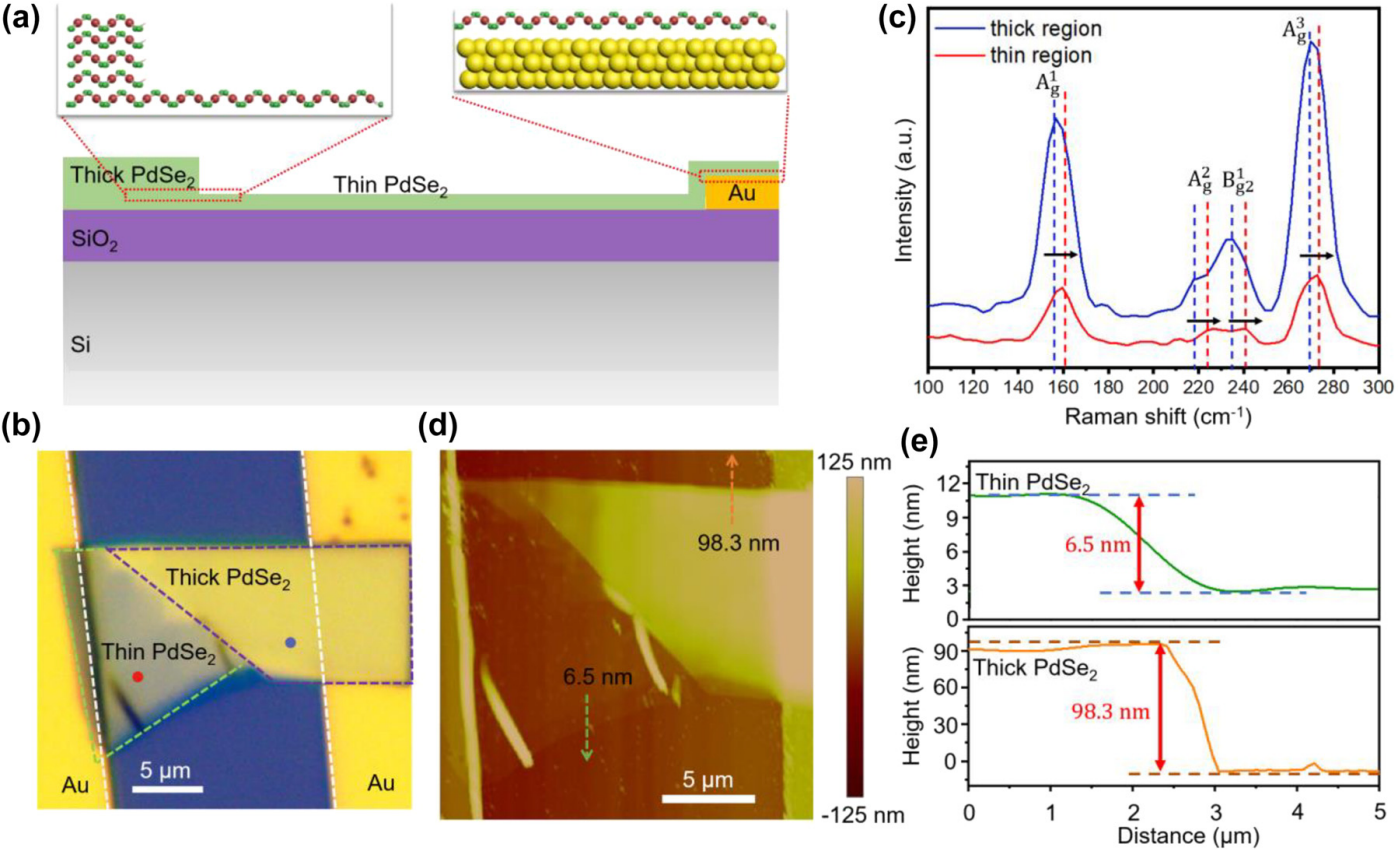
Structure characterization of the photodetector with asymmetric vdWs contacts. (a) The schematic diagram of a PdSe_2_ photodetector. (b) The optical image of the PdSe_2_ photodetector with asymmetric vdWs contacts. The scale bar is 5 μm. (c) The Raman spectra of the PdSe_2_ nanoflake measured at the blue and red points marked in (b). The thin and thick PdSe_2_ have different Raman peak positions corresponding to ${\text{A}}_{\text{g}}^{1}$ , ${\text{A}}_{\text{g}}^{1}$ , ${\text{B}}_{\text{g}2}^{1}$ , and ${\text{A}}_{\text{g}}^{3}$ modes, which referred to a “blue-shift” labeled by the black arrows. (d) The AFM image of the photodetector with non-uniform thickness across the channel. The scale bar is 5 μm. (e) The corresponding thickness measurements of the PdSe_2_ nanoflake from the AFM image, with a thickness of 6.5 nm for the thin part and 98.3 nm for the thick part. The measurement directions are illustrated as the green and orange dotted arrows presented in (d).

**Device architecture of the PdSe**_**2**_ **photodetector.** As illustrated in Figure 1(a), the PdSe_2_ nanoflake composed of a thin region and a thick region was fabricated on a Si/SiO_2_ substrate through mechanical exfoliation. For the thick region of the nanoflake with multilayer PdSe_2_, a layered crystal structure can be observed with a strong vdWs force combining each layer [48]. Here, the thin PdSe_2_ nanoflake works as the active material, which is asymmetrically vdWs contacted with a thick PdSe_2_ flake and a bottom Au electrode (10 nm Ti/50 nm Au). The optical microscope image of the fabricated photodetector with asymmetric vdWs contacts was presented in Figure 1(b). The apparent color difference indicates the coexistence of thin and thick regions in this nonuniform-thickness PdSe_2_ nanoflake. In addition, the Raman spectrum measurement was carried out to study the thickness of the nonuniform PdSe_2_ flake. As shown in Figure 1(c), four Raman intensity peaks of the thin PdSe_2_ (red line) and thick PdSe_2_ (blue line) were observed corresponding to ${\text{A}}_{\text{g}}^{1}$ , ${\text{A}}_{\text{g}}^{1}$ , ${\text{B}}_{\text{g}2}^{1}$ , and ${\text{A}}_{\text{g}}^{3}$ Raman modes. It can be noticed that all Raman peaks of the thin PdSe_2_ region are blue-shifted (deviate a little to the higher frequency) compared with the thick region, which can be ascribed to the strong interlayer coupling of PdSe_2_ nanoflake and special layer hybridization [49]. The thin and thick PdSe_2_ have different bond lengths between the atoms in each layer, leading to the slight modification of a vibration mode. The Raman spectra exhibit a blue shift phenomenon which verifies the coexistence of thin and thick regions in this PdSe_2_ nanoflake [50]. Furthermore, the thickness of the nonuniform PdSe_2_ nanoflake was exactly determined using atomic force microscopy (AFM) as presented in Figure 1(d). The line profile shown in dashed green and orange arrows was utilized to measure the thicknesses of the two different regions of the nonuniform PdSe_2_ nanoflake, and the measured thicknesses are 6.5 nm (about 10 layers) and 98.3 nm for the thin and thick regions, respectively (Figure 1(e)).

**Figure 2:**
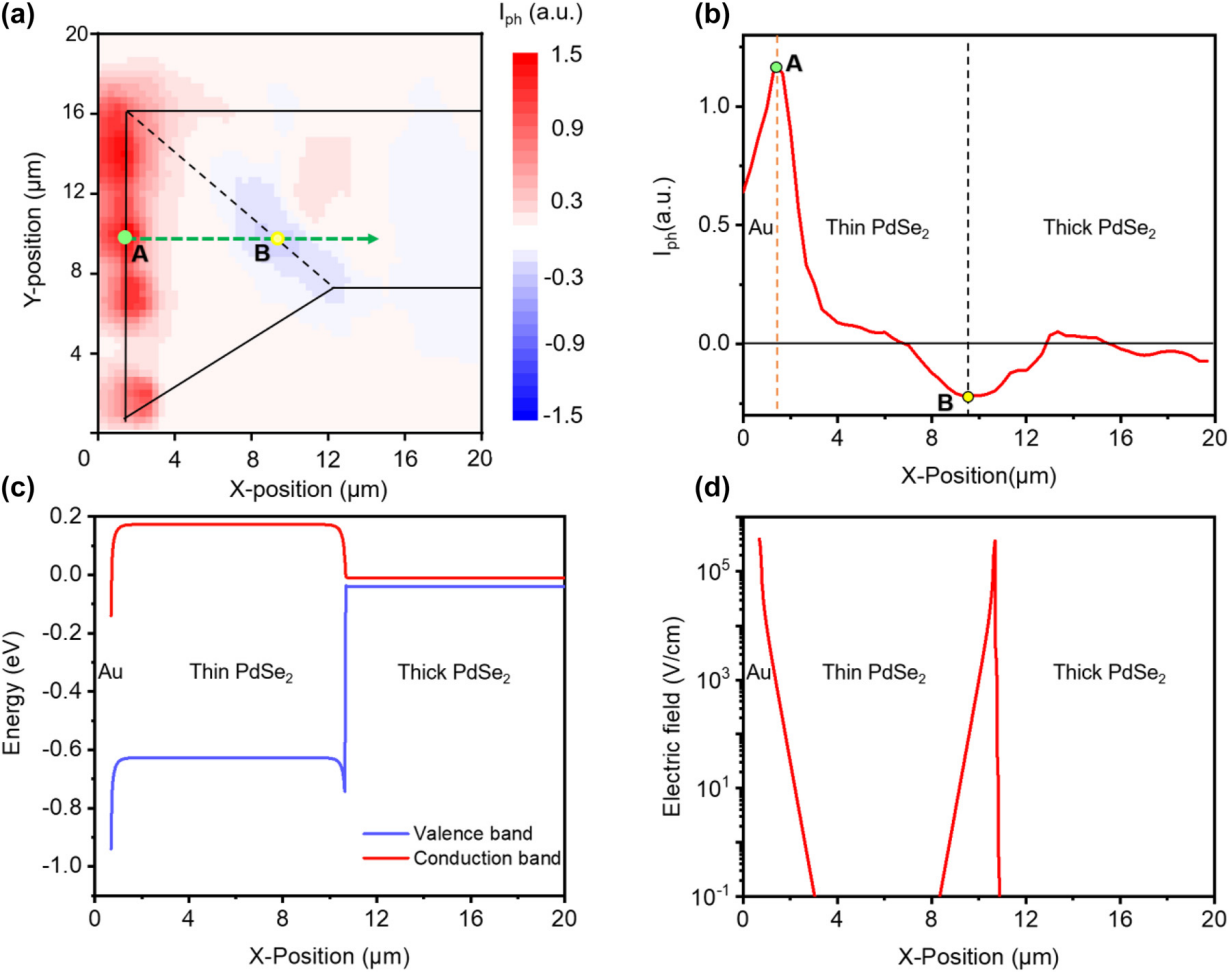
The photoresponse mechanism of the PdSe_2_ photodetector. (a) The local laser-induced photocurrent mapping exhibits ambipolar photocurrent-generation origins in the “thin-Au” contact and “thin–thick” interface. The black dashed line indicated the whole outline of the PdSe_2_ nanoflake in the channel. (b) The photocurrent distribution measured along the green dashed line in (a) presents an asymmetric ambipolar photocurrent existing along the channel. (c) The simulation of the bandgap of the thin PdSe_2_ photodetector with asymmetric vdWs contacts. (d) The simulated electric field distribution along the device channel.

**Photoresponse mechanisms of the PdSe**_**2**_ **photodetector**. To figure out the photoresponse mechanism of the PdSe_2_ photodetector with asymmetric vdWs contacts, a local laser-induced photocurrent mapping was carried out and presented in Figure 2(a). The distribution of photocurrent (*I*_ph_) excited by a 532 nm laser radiation was illustrated at zero bias. The black dashed line indicates the interface between the thick and thin PdSe_2_ nanoflakes. As shown in Figure 2(a), positive photocurrents appear in the contact region of the thin PdSe_2_ region and the Au electrode, while negative photocurrents are generated in the lateral homojunction region between the thin and the thick PdSe_2_ nanoflakes. In addition, a line profile curve of the photocurrent along the green dashed arrow in the device channel is extracted and shown in Figure 2(b). Points A and B are corresponding to the largest positive and negative photocurrents as marked in Figure 2(a). The photocurrent near the “thin PdSe_2_-Au contact” region (point A) has a much higher absolute value than the “thin PdSe_2_-thick PdSe_2_ contact” region (point B). As a result, under a global illumination, there will be a positive net photocurrent because of the majority contribution from the Schottky region between the thin PdSe_2_ and Au electrode. This photoresponse mechanism in our device is different from previously reported thickness-based lateral heterojunction photodetectors [51].

To clarify the photoresponse mechanism of the photodetector based on asymmetric vdWs contacts, the energy band diagrams were calculated and analyzed (Supplementary Note 1 and Figure S1a). Based on the theoretical bandgap and the reported experimental parameters of PdSe_2_, the bulk PdSe_2_ can be treated as a semimetal material with a zero bandgap (∼0.03 eV), thus an Ohmic contact was realized between the Au electrode and thick PdSe_2_ [52]. And no potential barrier in this region means that there is no existing separation of electron-hole pairs in this area and no contribution to the final output photocurrent, which can be observed directly in Figure 2(a). In contrast to the zero bandgaps of the bulk PdSe_2_, the 10-layer PdSe_2_ has a bandgap of about 0.8 eV [53]. As we all know, the Schottky barrier height will influence the efficiency of photoresponse based on the photovoltaic effect [54, 55]. The band diagram of the 10-layer and bulk PdSe_2_ was calculated after contact equilibrium (Figure S1b). After the contact, a built-in potential (∼0.41 eV) was formed between the electrode and 10-layer PdSe_2_, while a smaller built-in potential (∼0.38 eV) was generated between the 10-layer and bulk PdSe_2_ (See Supplementary Information Note 1). In addition, to better understand the response origin of this photodetector with asymmetric vdWs contacts, the energy band diagram of this PdSe_2_ device was simulated, as presented in Figure 2(c). Two asymmetric Schottky barriers are formed at two asymmetric vdWs contacts owing to the different work function between Au and thick semimetal PdSe_2_ nanoflake. Moreover, the simulated electric field along the channel of the PdSe_2_ device is depicted in Figure 2(d). And two electric field peaks can be observed in the “Au-thin PdSe_2_” contact region and the “thin-thick PdSe_2_” contact region. The higher value (4 × 10^5^ V cm^−1^) of the electric field in the “Au-thin PdSe_2_” interface is arising from the higher built-in potential as discussed above. Once the local laser illuminates the “thin PdSe_2_-Au” contact region, a strong photocurrent can be collected, which indicates that a separation of the electron-hole pairs happened in this region. Then the electrons diffused to the left electrode and were collected. When the laser was localized on the “thin-thick PdSe_2_” contact region, there was also a separation of electron-hole pairs, and the electrons diffused to the thick PdSe_2_ region. Therefore, a reversed and weaker photocurrent can be measured, which matched the experimental results in Figure 2(a). According to this band theory, the detailed working mechanism can be attributed to the asymmetric photovoltaic effect in this device originating from the asymmetric vdWs contacts, which is dominated by the Schottky junction between the Au electrode and thin PdSe_2_. This enables the photodetector to realize a fast photoresponse with zero applied bias. In addition, a PdSe_2_/PdSe_2_ homogenous junction device (Figure S7a) was fabricated and tested under a 532 nm laser, with no apparent photocurrent generated (Figure S7b). This further confirmed that the dominant photoresponse should be ascribed to the photovoltaic effect in the contact region between the thin PdSe_2_ and the Au electrode.

Besides, it is notable that PTE effect can be distinguished from the photoresponse in this device, as the apparent photocurrent happens only when the laser illuminates the interface of the junction region. It is a typical feature of the photovoltaic effect, while the PTE current can be located in a larger area, not only limited to the junction regions [56]. And we can also exclude the possible photocurrent generated from the thin PdSe_2_ in the middle of the two contact regions as the main photoresponse mechanism in a Au/thin PdSe_2_/Au device has been studied to be photoconductive effect which needs an external bias to generate a photocurrent [3]. And a strong photocurrent generated under zero bias between the electrodes can exclude the photoconductive effect from this device since a source-drain voltage is necessary for the photoconductive photodetectors to generate output photocurrent.

**Broadband photodetection characterization of the PdSe**_**2**_ **photodetector**. To further evaluate the broadband photodetection performance of this PdSe_2_ photodetector, a systematical investigation was scheduled by using lasers with different wavelengths ranging from visible (532 nm) to near-infrared (1470 nm). All the measurements were carried out in a room-temperature environment. It is noticeable that the diameters of the laser spots corresponding to various wavelengths are all much larger than the size of the device. This means that a global illumination was realized during the whole testing procedure, with the final output signal being a combination of the photoresponse in the asymmetric vdWs contact regions.

**Figure 3:**
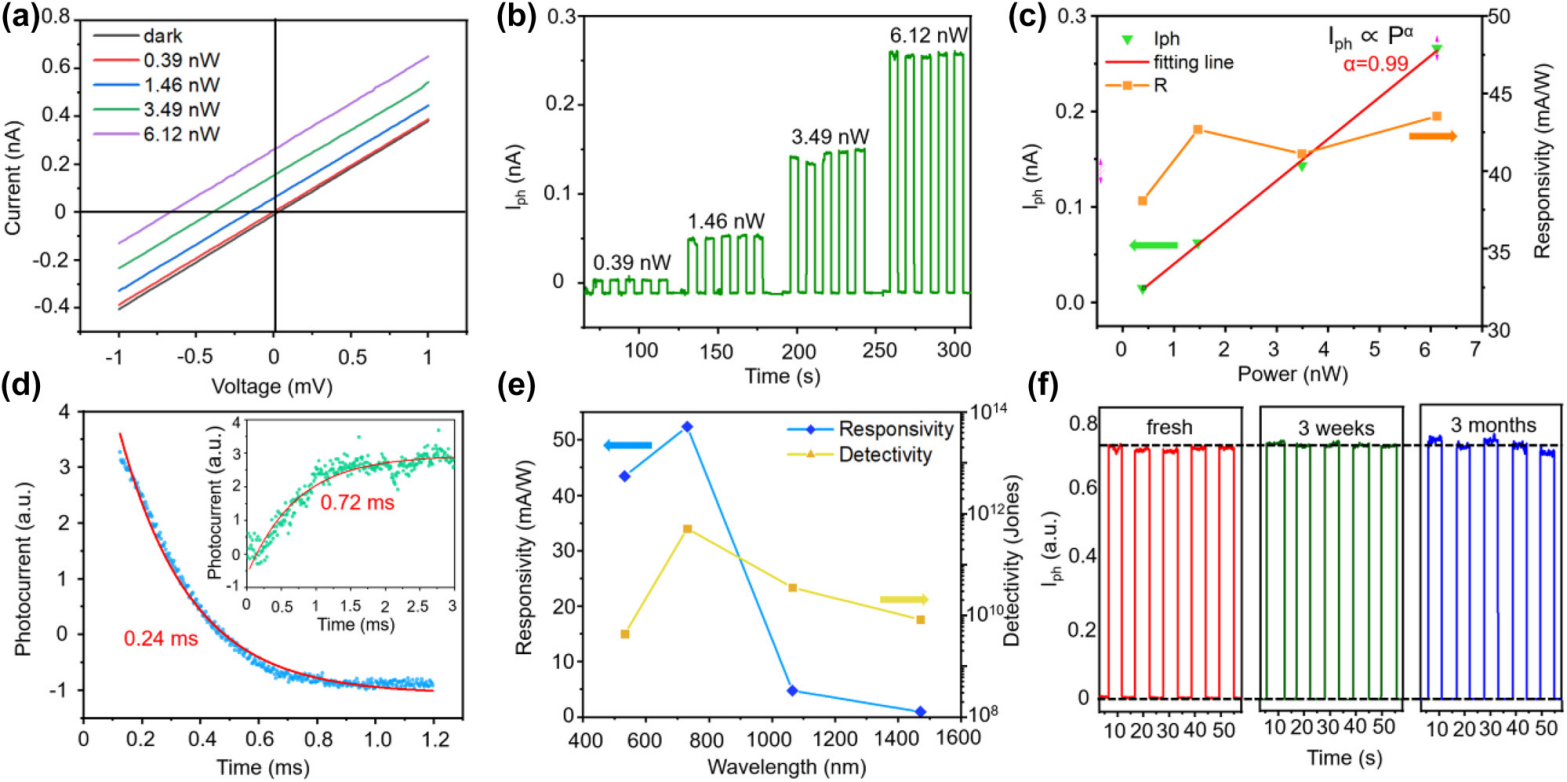
The performance of the PdSe_2_ photodetector. (a) The *I*–*V* curves of the photodetector under dark and global illumination with a 532 nm laser. The power changes from 0.39 nW to 6.12 nW. (b) Time dependence of photoresponse under pulsed laser radiation with various powers ranging from 0.39 nW to 6.12 nW at zero bias. (c) The power dependence of photocurrent and responsivity under a wavelength of 532 nm. The fitting line is the red solid line plotted by the power law, demonstrating a linear relationship between photocurrent and incident light power. (d) The illustration of the rise time (0.72 ms) and decay (0.24 ms) time of the photoresponse, indicates a fast response speed. All data were measured under a 532 nm laser with a fixed power. (e) The spectral responsivity and detectivity of the PdSe_2_ photodetector as a function of the incident laser wavelength. (f) The photocurrent as a function of the time measured with a pulsed 532 nm laser. And the same test was repeated after 3 weeks and 3 months respectively, indicating the long-term stability of this photodetector.

The *I*–*V* curves of this PdSe_2_ photodetector are shown in Figure 3(a), measured under dark and a 532 nm laser with incident power changing from 0.39 nW to 6.12 nW. The linearity of the *I*–*V* curves demonstrate that a low barrier exists in both the “electrode-thin PdSe_2_” contact region and the “thin PdSe_2_-thick PdSe_2_” contact region. Besides, a slight rectification characteristic can be observed indicating the asymmetric Schottky barriers in this device (Figure S2, Supplementary Information), which is consistent with the analysis of the energy band diagram and the photoresponse mechanism (Figure 2). Then, the temporally resolved photoresponses of the device at zero bias were measured as shown in Figure 3(b). During the test, a chopper was utilized to modulate the on/off states of the laser with a period of 5 s. As presented in the curve, a repeatable and increasing photoresponse excited by enhanced light powers can be observed. Based on the experimental results, *I*_ph_, responsivity (*R*), and specific detectivity (D^*^) can be calculated by the following formulas:

(1) $$I_{\text{ph}}=I_{\text{light }-\text{ on}}-I_{\text{light }-\text{ off}}$$

(2) $$R=\frac{I_{\text{ph}}}{P}$$

(3) $$D^{*}\approx \frac{R\sqrt{A}}{\sqrt{2qI_{\text{dark}}}}$$

where *I*_ph_ indicates the photocurrent, *P* represents the effective light power at a device, A is the active area of the photodetector, *q* is the elementary charge, and *I*_dark_ is the dark current. The calculated photocurrent increases almost linearly with the increased light power and the responsivity changes from 38 to 43 mA/W as shown in Figure 3(c). And the relationship between incident light and photocurrent can be fitted by law power law *I*_ph_ ∝ *P*^*α*^, where *P* is the actual incident power illuminated on the photodetector and *α* is the ideal factor. And through fitting the photocurrent corresponding to the incident power, the calculated ideal factor is 0.99. And based on the responsivity *R* = *I*_ph_/*P*, almost constant responsivity can be expected with the increase of incident power. It is noticeable that we measured the photoresponse in a small power range which is in the linear dynamic range (LDR) of the photodetector, and a linear relationship between *R* and incident laser power is demonstrated. As the laser power goes beyond the LDR, a strongly-power-related *R* can be measured [57, 58]. Figure 3(d) shows a detailed measurement of the rise/decay time. By fitting the time-resolved photoresponse using the exponential decay function, the rise/decay time with 0.72 ms/0.24 ms can be obtained directly. The result demonstrates a fast response speed in this PdSe_2_ photodetector ascribed to the photovoltaic effect, which is comparable to the traditional 2D materials-based photodetectors [3, 26, 59].

To further characterize the broadband photodetection capability of this photodetector, another laser source with a wavelength of 1064 nm was utilized as a light source, as shown in Figure S3, Supporting Information. The *I*–*V* curves of the photodetector under a 1064 nm laser with various incident power ranging from 8.8 nW to 52.3 nW are presented in Figure S3a, with a similar linear characterization compared with the result measured under a 532 nm laser. Then the photoresponse of the photodetector under pulsed laser radiation with various powers from 8.8 nW to 52.3 nW was collected, as illustrated in Figure S3b. With the increase of the incident light power, the corresponding photocurrent increases from 0.05 nA to 0.3 nA, presenting a nearly linear relation with the illumination power. And the calculated photoresponsivity changes from 3.55 mA/W to 5.12 mA/W, demonstrating a high sensitivity to near infrared illumination (Figure S3c). Besides, to verify the photoresponse of this device towards the light with other wavelengths, two laser sources with a wavelength of 730 nm and 1470 nm were also utilized to determine the sensitivity of this photodetector, as demonstrated in Figure S4, Supporting Information. The photoresponse under pulsed laser radiation is shown in Figure S4a (730 nm) and Figure S4b (1470 nm), with an incident light power of 48.9 nW and 33.4 nW, respectively. And a strong photoresponse with a generated photocurrent of 0.65 nA under the 730 nm light can be measured, showing the great ability to detect visible light by this photodetector. And a ∼25 pA photocurrent illuminated by a 1470 nm laser could be collected, demonstrating the broadband spectral response ability of this photodetector.

Based on the photoresponse investigation of this photodetector, the calculated *R* and D^*^ are presented in Figure 3(e), as a function of the illumination wavelength. Both the peaks of responsivity of 53 mAW^−1^ and detectivity of 5.17 × 10^11^ Jones located at the 730 nm wavelength are achieved. However, there are other types of noise contributing to the total device of the photodetector beside of the dark current, like thermal noise or 1/*f* noise. In order to further study the noise in the device, we calculated the thermal noise in our photodetector based on the formula $i_{j}=\sqrt{\frac{4k_{\text{B}}t}{R}}$ , where *k*_B_ is Boltzmann constant, *t* is the temperature and *R* is the resistance of the device. And the calculated thermal noise is about 7.61 × 10^−14^ A which is three orders of magnitude smaller than the dark current with a larger value of 1.54 × 10^−11^ A. We also measured the 1/*f* noise spectra at a low frequency region, and the 1/*f* noise current proved to be much smaller than the dark current (Figure S8). Therefore, for simplicity, the shot noise from dark current density is assumed to be the dominant contribution to total noise in our device [60]. By comparing with the performances of recently developed photodetectors based on photovoltaic effect, this broadband PdSe_2_ photodetector shows attractive and comparable photoresponse properties (See Supporting Information Table S1 for detail) [3, 26, 46, 54, 59, 61], [62], [63], [64]. Besides, the durability and stability of this photodetector were also investigated. As shown in Figure 3(f), the ideal photoresponse stability over a long duration of time ranging from the fresh to 3 months has been observed. We also conducted a repeated illumination test over 360 circulations based on a homemade testing system (Figure S5a, Supporting Information). A chopper with a fixed frequency was applied to control the on/off status of the illumination. And good repeatability of this photodetector was also confirmed (See Supporting Information Figure S5b for detail).

**Figure 4:**
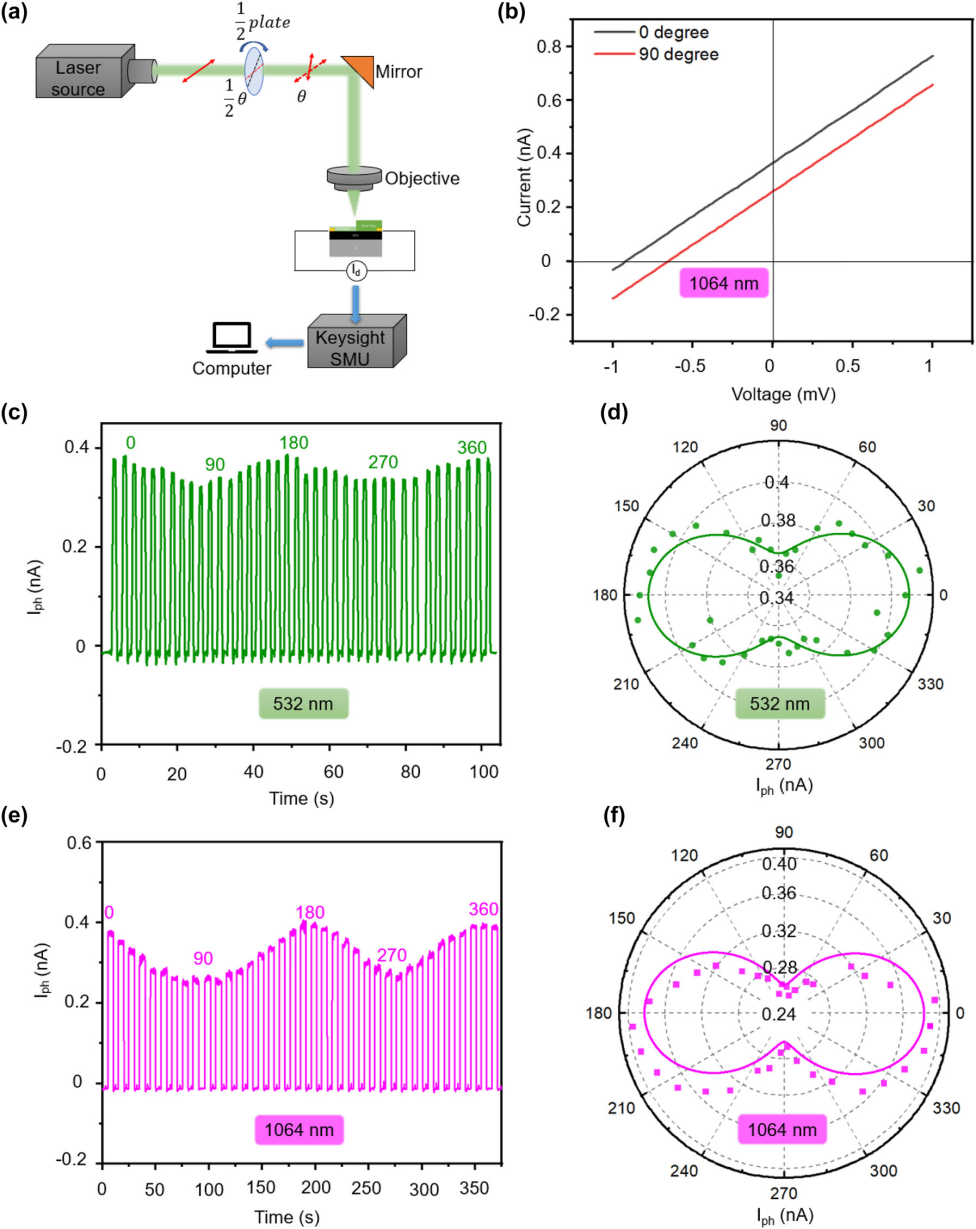
Polarized photoresponse of the PdSe_2_ photodetector. (a) Schematic illustration of a photodetector measurement system for analyzing linear polarization sensitive response. (b) The *I*–*V* curves of the device under 1064 nm illumination with linear polarization angles of 0° and 90°. (c) and (e) The linear-polarization-angle dependent photocurrents under 532 nm (c) and 1064 nm (e) light illuminations. Here 0° corresponds to the angle of the incident light when the laser polarization direction is aligned with the *x*-direction of the asymmetric-thickness PdSe_2_. (d) and (f) The generated photocurrent as a function of the polarization angle of the laser. All the dots in (d) and (f) are fitted with the (*a* + *b*cos 2*θ*) function.

**The polarization sensitivity of the PdSe**_**2**_ **photodetector**. 2D materials were recently discovered to have attractive anisotropic properties [65, 66], and the photodetectors based on them established high performance in polarization light detection. Typically, non-polarized photodetectors are typically solely sensitive to the intensity and wavelength of the input light while the polarized photodetectors can directly detect polarized light in addition to intensity and wavelength. Using polarization-sensitive photodetectors, the information and status of the polarized incident light can be distinguished and extracted [67, 68]. And applications such as optical communications, optical switching, polarization sensing systems, and optical radar all depend on the ability of the photodetector to detect polarized light [69]. Among the different 2D anisotropic materials, PdSe_2_, coupled with an anisotropic crystal structure, can enable the device to be sensitive to the polarized incident light. To identify the polarization light response properties of this PdSe_2_ photodetector, the generated *I*_ph_ in this device under linear polarized light was measured and collected. The measurements were carried out through changing the linear polarization angle of the incident light based on a homemade polarization measurements setup, as illustrated in Figure 4(a). Paralleled *I*–*V* curves briefly indicate that this PdSe_2_ photodetector output different electrical signals depending on light with different polarization angles. Then a systemic investigation of the polarized photoresponse was demonstrated. As presented in Figure 4(c) and (e), the *I*_ph_ was collected at the incident laser with wavelengths of 532 nm and 1064 nm. And a periodic change can be observed corresponding to the varying polarized angle. Besides, the polar coordinates of the *I*_ph_ under 532 nm and 1064 nm polarized light were plotted, as shown in Figure 4(d) and (f), respectively. A two leaves-shape of the polarization-dependent curves fitted by the (*a* + *b*cos2*θ*) function was illustrated. And the ratio of anisotropy ellipses can be calculated to be 1.11 and 1.62 at 532 and 1064 nm, respectively. These results prove that this PdSe_2_ device can achieve a broadband polarization light response, which makes it possible to work in practical applications like polarization imaging. Besides, a discrepancy in the ratio of anisotropy under 532 nm and 1064 nm laser can be confirmed. This can be explained by the fact that the polarized absorption of PdSe_2_ is strongly affected by the incident wavelength, which will lead the ratio of anisotropy to be different when illuminated by lasers with different wavelengths [70, 71].

**Figure 5:**
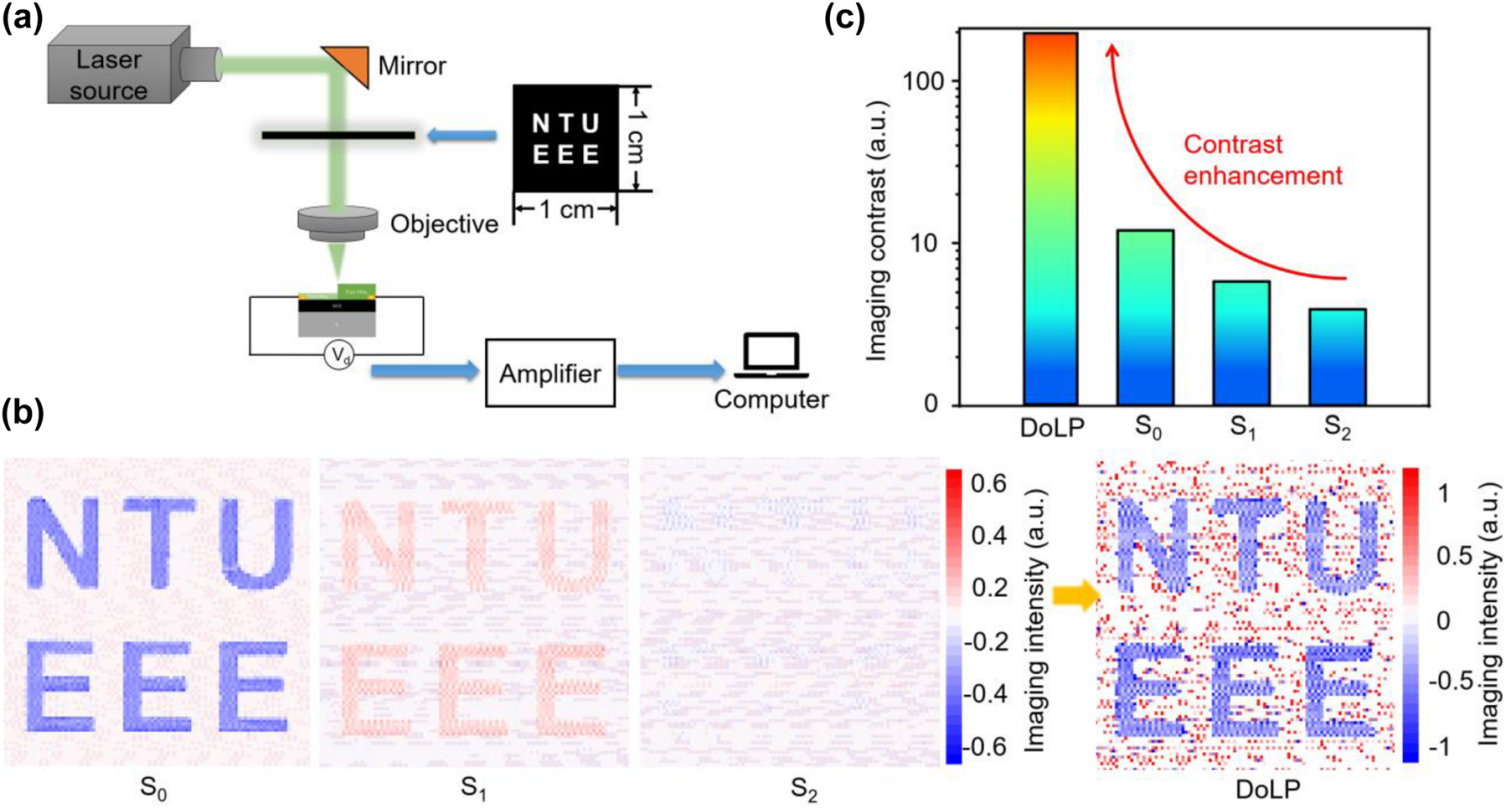
Polarization imaging measurements using the PdSe_2_ photodetector. (a) Schematical demonstration of the setup of the polarized imaging test system. (b) The calculated normalized contrast constant of *S*_0_ and DoLP images. (c) The calculated *S*_0_, *S*_1_, *S*_2_, and the final DoLP results with a large contract constant with the 8-neighbors contrast calculation method, indicating the extraordinary polarization detection ability of this device.

**Polarization imaging**. Realistic imaging requires both high sensitivity and detectivity [72], and high-quality polarized imaging relies on the polarized photoresponse of photodetectors. The high sensitivity, fast photoresponse speed, and excellent polarization photoresponse demonstrated in this work endow this device with potential to realize polarized imaging. Based on the outstanding polarized photoresponse, high-quality polarization imaging with an enhanced imaging contrast can be expected in this device. To further investigate the polarization imaging capabilities of this photodetector, a homemade imaging test setup was designed as illustrated in Figure 5(a). The linear polarization light directly illuminated the fabricated photodetector and the output photovoltage was collected and amplified by an amplifier, and the final polarized images were processed and presented in a computer. A metallic object with “NTU EEE” letters was utilized as the pattern, and the polarized imaging was acquired with the photodetector by changing the pattern location. The imaging results under linear-polarized light with polarized angles of 0°, 45°, 90°, and 135° were obtained, as shown in Figure S6a, Supporting Information. Then, the spatial distribution of degree of linear polarization (DoLP) was calculated by Eqs. (4)–(7), (Figure S6b, Supporting Information),

(4) $$DoLP=\frac{\sqrt{S_{1}^{2}+S_{2}^{2}}}{S_{0}}$$

(5) $$S_{0}=I_{0{}^{\circ}}\left(x,y\right)+I_{90{}^{\circ}}\left(x,y\right)$$

(6) $$S_{1}=I_{0{}^{\circ}}\left(x,y\right)-I_{90{}^{\circ}}\left(x,y\right)$$

(7) $$S_{2}=I_{45{}^{\circ}}\left(x,y\right)-I_{135{}^{\circ}}\left(x,y\right)$$

where *I*(*x*,*y*) refers to the generated *I*_ph_ measured at a polarized angle of *θ* [72, 73]. The calculated imaging results including *S*_0_, *S*_1_, *S*_2_, and DoLP were presented in Figure 5(b). Furthermore, the imaging contrasts of the *S*_0_, *S*_1_, *S*_2_, and DoLP images were calculated based on the 8-neighbors contrast calculation method and presented in Figure 5(c). The results show that an enhanced imaging contrast for the target was observed in the DoLP image compared with *S*_0_, *S*_1_, and *S*_2_. This demonstrated that this PdSe_2_ photodetector can be a promising candidate for polarization imaging applications [72].

## 3 Conclusions

In summary, a broadband, polarization-sensitive, and self-powered PdSe_2_ photodetector with asymmetric vdWs contacts was realized. A strong asymmetric photovoltaic effect was generated between the Au-thin PdSe_2_ and the thin-thick PdSe_2_ contact regions, which supports the self-powered photodetection. This self-powered photodetector exhibits a promising photoresponse for the visible-near infrared light in a broad region from 532 nm to 1470 nm, with a peak responsivity over 53 mA/W and a high detectivity of 5.17 × 10^11^ Jones at 730 nm. In addition, benefiting from the high operation speed of photovoltaic effect, an attractive response speed with a rise/decay time of about 0.72 ms/0.24 ms is achieved. Besides, due to the excellent anisotropic optical properties of the PdSe_2_ nanoflake, this photodetector exhibits a polarization-sensitive photoresponse with an anisotropic ratio of 1.11 and 1.62 at 532 nm and 1064 nm, respectively. Considering the fast response speed, high sensitivity, and polarization sensitivity, the polarization imaging using our proposed device is further demonstrated, and a contrast-enhanced degree of linear polarization imaging is realized. All these results show the tremendous potential of this type of device in achieving a self-powered, broadband, and polarization-sensitive photoresponse, with a potential application in polarization imaging.

## 4 Experimental section

**Device Fabrication**. The PdSe_2_ flakes were naturally mechanically exfoliated from the bulk PdSe_2_ material. The SiO_2_/Si wafer was treated as a substrate, and it was washed in an ultrasonic cleaner with isopropanol, acetone, ethanol, and deionized water separately in advance. The conventional UV lithography and e-beam evaporation techniques were applied to form two paralleled electrodes (10 nm Ti/50 nm Au) on the Si/SiO_2_ substrate. Then, the previously exfoliated PdSe_2_ nanoflake with non-uniform thickness was transferred on the substrate and aligned with the Au electrodes by a polydimethylsiloxane (PDMS) assisted dry transfer method. The thin and thick regions of the PdSe_2_ nanoflake were contacted with these two electrodes, respectively.

**Material Characterization**. The optical image of the sample was characterized by a Nikon optical microscope. A WITec (Alpha 300) micro-Raman spectrometer system was utilized to measure the Raman spectra of the PdSe_2_ nanoflake pumped by a 532 nm laser source. And the exact thicknesses of the two different parts of this PdSe_2_ nanoflake were measured by atomic force microscopy (Bruker Dimension Icon).

**Device Characterization.** For the spatial photocurrent mapping, the Raman system with a scanning micromotion platform was applied, excited by a focused 532 nm laser scanning over the device. And during the mapping test, an amplifier was coupled with the device to amplify and extract the output signal. In the electrical measurements of the device under dark and illumination, a Keysight, B2912A digital source meter owning two highly sensitive channels were used, where t the output current and photovoltage were collected at the same time. And for the transient photoresponse test, a timer mode with a time resolution of 5 μs was set in the source meter and the laser source was modulated by a chopper. Four different laser sources with fixed laser wavelengths of 532, 730, 1064, and 1470 nm (MIDL-III-532, 730, 1064, and 1470) were applied to illuminate the active region of the photodetector. And the diameters of the laser spot were ∼5.6, 5.8, 6.3, and 9.2 mm for the 532, 730, 1064, and 1470 nm lasers. The laser power of these laser sources was measured by a Thorlabs, PM100D power meter.

**Polarized light detection measurements**. In the polarized light detection measurements, a half-wave plate was employed to modulate the polarization angle. With changing rotation angle of the plate, the polarization angle of incident light can be modulated. The laser polarization direction initially parallels the *x*-axis of our devices, and the rotation step of the half-wave plate is 5°, which corresponded to a step of 10° for the polarization angle. Then the laser signal with different polarization angles was detected by the photodetector and the output photocurrent was collected by the source meter.

**Polarization imaging**. The polarization imaging was demonstrated by a homemade scanning system. The light source we chose is a single-wavelength laser with a fixed wavelength of 532 nm. In the polarized light imaging measurements, a half-wave plate was put between the laser source and the imaging target, to simulate the linear polarized state of the target. The polarized angle of the laser was set to be 0°, 45°, 90°, and 135°. All the corresponding polarized images were collected by the homemade imaging system. There are two step motors (along the *x*-axis and *y*-axis) controlling the location of the text pattern. By changing the pattern location, a photocurrent signal is acquired for each pixel.

## Supporting information

The calculation and simulation of the energy band diagram, the *I*–*V* current under different applied voltages, the investigation of the photoresponse under laser at 1064 nm, the photoresponse under laser at 730 nm and 1064 nm, the photoresponse stability of PdSe_2_ photodetector, the comparison between the recent self-power photodetectors based on 2D materials, the polarized imaging result and the calculating method of DoLP.

## Supplementary Material

Supplementary Material Details

## Supplementary Material

The article contains supplementary material (https://doi.org/10.1515/nanoph-2022-0660).
