# Supplementary material for: A broadband, self-powered, and polarization-sensitive PdSe2 photodetector based on asymmetric van der Waals contacts
Source: Nanophotonics. 2023 Jan 11;12(3):607–18. doi: 10.1515/nanoph-2022-0660 (PMC11501662; doi:10.1515/nanoph-2022-0660)
Supplement: Supplementary file 1 — Supplementary Material Details [file j_nanoph-2022-0660_suppl.docx]

**Supporting information**

A Broadband, Self-Powered, and Polarization-Sensitive PdSe2 Photodetector Based on Asymmetric van der Waals Contacts

Xuran Zhang^1#^, Mingjin Dai^1#^, Wenjie Deng^1,3^, Yongzhe Zhang^3^, Qi Jie Wang^1,2, *^

^1^School of Electrical and Electronic Engineering, 50 Nanyang Avenue, Nanyang Technological University, Singapore 639798, Singapore

^2^School of Physical and Mathematical Sciences, Nanyang Technological University, Singapore 637371, Singapore

^3^Key Laboratory of Optoelectronics Technology, Ministry of Education, Faculty of Information Technology, Beijing University of Technology, Beijing 100124, China

^#^Equal contribution

*Email: [qjwang@ntu.edu.sg](mailto:qjwang@ntu.edu.sg)

**Supplementary Note 1. Band structure calculations of lateral PdSe_2_ homojunction.**

Firstly, the number of layers of the thin region of PdSe_2_ is estimated to be about 10 layers, and the bandgap of a 10-layer PdSe_2_ nanoflake is approximately 0.8 eV.^[1]^ The electron affinity of PdSe_2_ is 5.12 eV.^[2, 3]^

The carrier concentration of the nanoflake can be calculated by the formula proposed by Huang:^[4]^

$n_{2d}=I_{\mathrm{DS}}\times C_{i}/\left( e\times\frac{\partial I_{\mathrm{DS}}}{V_{G}} \right)$(1)

where I_DS_ is the output current measured at zero gate voltage, C_i_ is the capacitance of a 300 nm SiO_2_ substrate used in this work, with a value of 1.15× 10^−8^ F/ cm^2^, e is the electron charge. We calculated that carrier concentration of this PdSe_2_ nanoflake to be 4.26×10^12^ cm^-3^.^[5]^

Then we can calculate the conduction band equivalent density of states based on the following equation:

$N_{C}=2\cdot\frac{\left( 2\pi m_{n}^{*}k_{0}T \right)^{\frac{3}{2}}}{h^{3}}$ (2)

where $m_{n}^{*}$ is the effective mass of electrons in the material, k_0_ is the Boltzmann constant, T is the temperature and N_C_ is the conduction band equivalent density of states. The effective mass of the commonly used PdSe_2_ nanoflake is 0.8 m_0_.^[2]^ Based on these factors, we figured out the conduction band equivalent density of states of the thin PdSe_2_ nanoflake is about 1.79×10^19^ cm^-3^.

And the Fermi level can be determined by the following equation:

$n_{0}=N_{C}\exp\left( \frac{E_{F}-E_{C}}{k_{B}T} \right)$ (3)

We substituted the value of N_C_ into formula (3) to get the final ${(E}_{C}-E_{F})$ to be 0.394 eV for the thin region of the PdSe_2_ nanoflake.


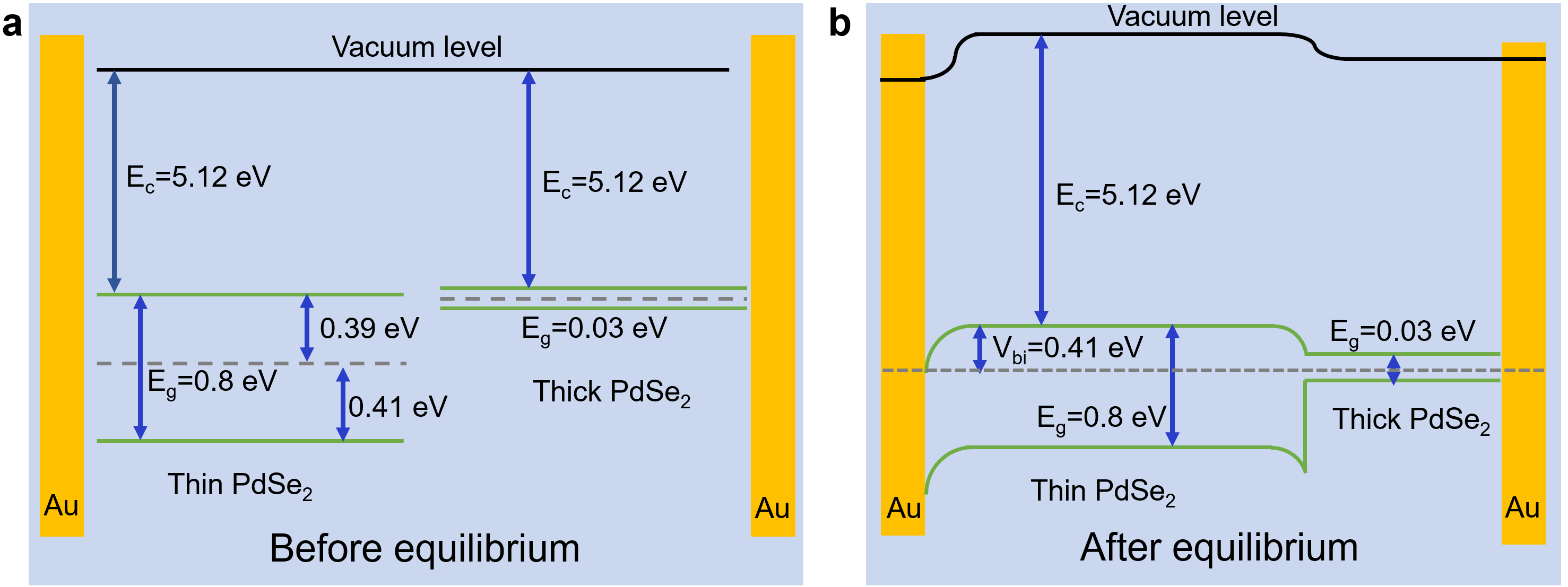


**Figure S1.** The energy band diagram of thin and thick PdSe_2_ (a) before equilibrium and (b) after equilibrium. The E_c_ and E_g_ are the electron affinity and bandgap of the material, respectively.


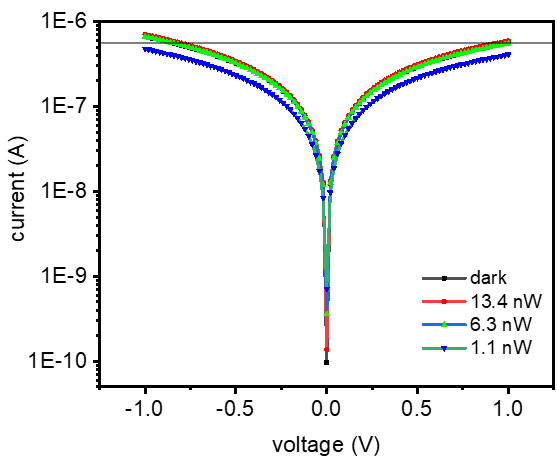


**Figure S2**. The output current as a function of the applied voltage between two electrodes, measured at the illumination by a 730 nm laser with incident power ranging from 0 to 1.1 nW. Labelled by the black line, a slight offset indicates that the photovoltaic effect dominates the photoresponse process.


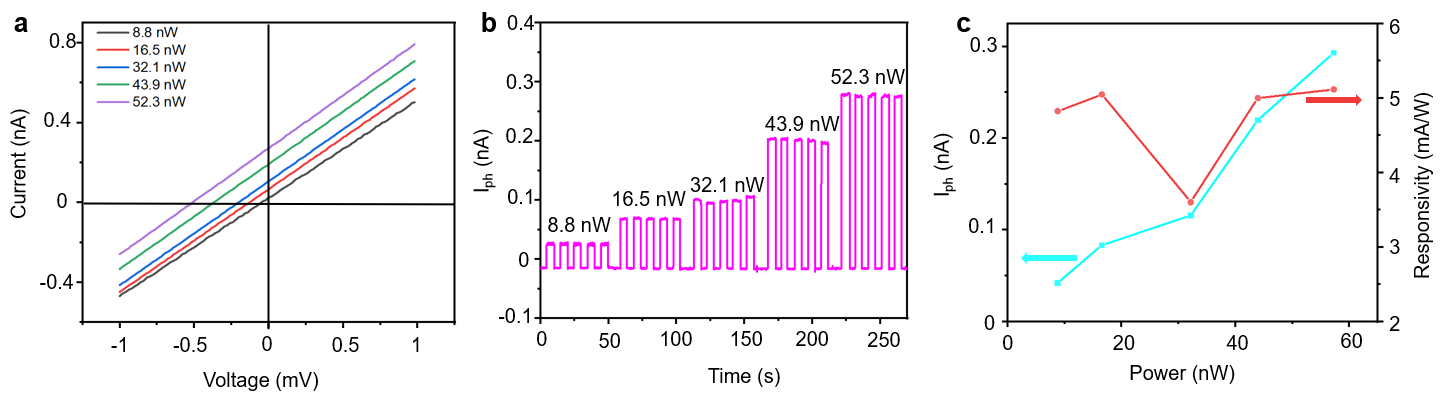


**Figure S3**. A systematical investigation on the photoresponse to 1064 nm laser of this photodetector. a) The I-V curve of the photodetector under dark and global illumination with a 1064 nm laser. The power changes from 8.8 nW to 52.3 nW. b) Photoresponse of the photodetector under pulsed laser radiation with various powers ranging from 8.8 nW to 52.3 nW. All the data were acquired at zero V_ds_ bias. c) The I-V curve of the photodetector under dark and global illumination with a 1064 nm laser. The power changes from 200 nW to 412.5 nW, indicating an apparent photovoltaic effect dominated in the photoresponse mechanism.


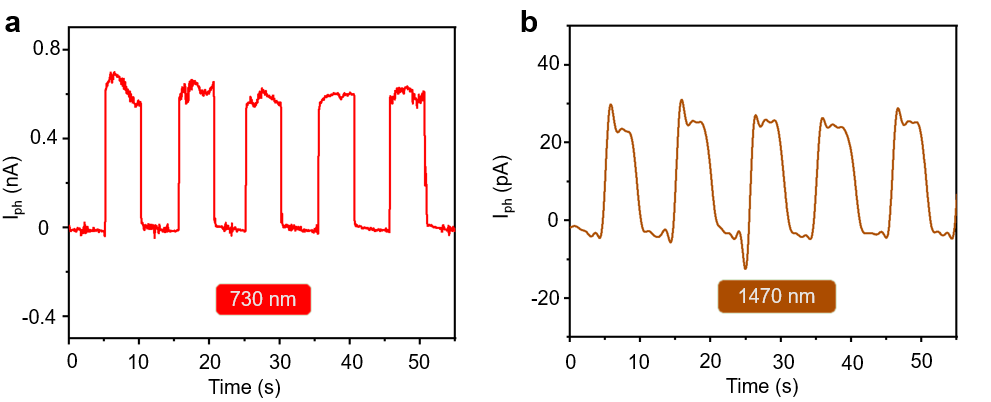


**Figure S4**. a) Photoresponse of the photodetector under pulsed laser radiation (wavelength of 730 nm) with power of 48.9 nW. All the data were acquired at zero V_ds_ bias. b) Photoresponse of the photodetector under pulsed laser radiation (wavelength of 1470 nm) with power of 33.4 nW. All the data were acquired at zero V_ds_ bias.


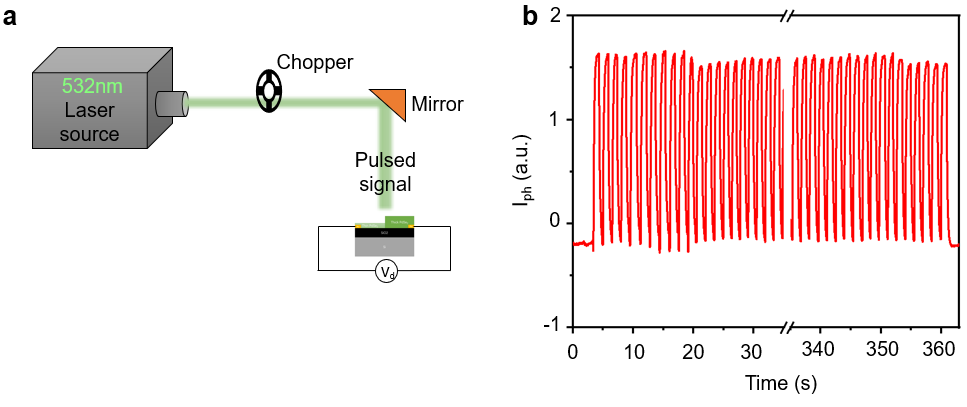


**Figure S5**. a) The schematical presentation of the experimental setup for the repeatability measurements of the device. b) Repeatability of the PdSe_2_ asymmetric-thickness photodetector.


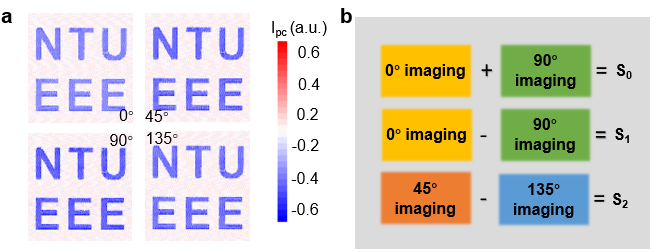


**Figure S6.** a) The detector-formed polarized imaging under linear-polarized light with polarized angle of 0°, 45°, 90°, and 135°. A text pattern NTUEEE was applied to helping generating images. b) A schematical diagram of the calculation of the DoLP result.


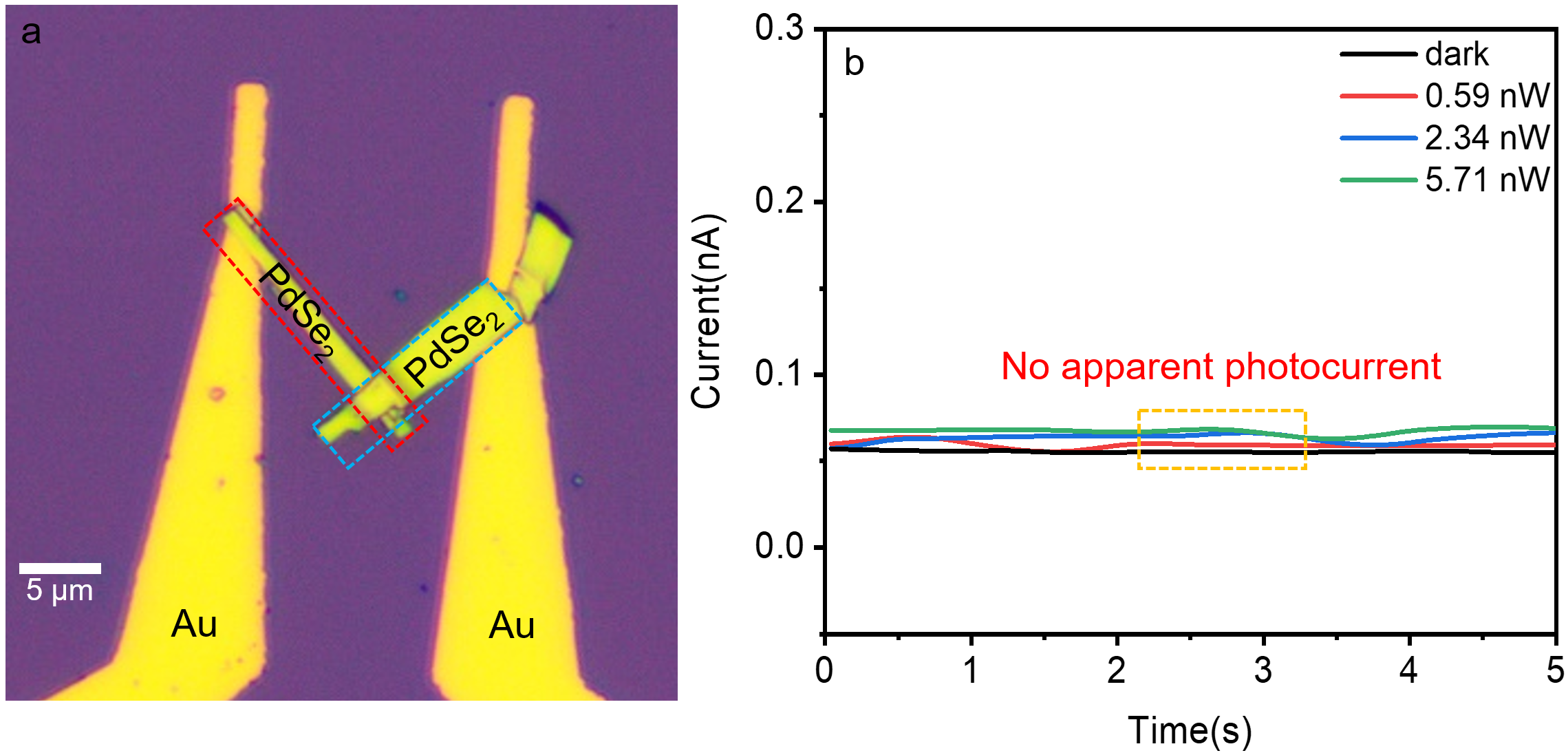


**Figure S7.** Tests of photoresponse in the PdSe_2_/PdSe_2_ homogenous junction device. a) The optical image of the PdSe_2_ homogenous junction device. The scale bar is 5 μm. b) Time dependence of photoresponse the PdSe_2_ homogenous junction device under pulsed laser radiation with various powers ranging from 0 nW to 5.71 nW at zero bias.

**Figure S8.** The measured low-frequency noise current spectra of the photodetector operated at zero bias.

**Table S1**. Performance comparison between the recent self-powered photodetectors based on photovoltaic effect.

| Material | Device structure | Spectral range (nm) | Responsivity (mA/W) | Detectivity (Jones) | Response speed | Ref |
| --- | --- | --- | --- | --- | --- | --- |
| BP-InSe | p–n heterojunction | 450-900 | 11.7 | - | 24 ms/32 ms | [6] |
| MoS_2_/CdTe | p–n heterojunction | 200-1700 | 36.6 | $6.1\times{10}^{10}$ | 43.7 μs/82.1 μs | [7] |
| MoS_2_/Ge | heterojunction | 350-1100 | 16.66 | - | - | [8] |
| PdSe_2_ | Schottky junction | 532 | 3.5 | - | 11 ms/6 ms | [9] |
| MoS_2_ | Schottky junction | 405-980 | 0.063 | $4.2\times{10}^{8}$ | $<\text{20 ms}$ | [10] |
| PdSe_2_/Si wafer | heterojunction | 1550 | 0.37 | $1.2\times{10}^{10}$ | 38 μs/44 μs | [11] |
| Au/MoS_2_/Mo_2_C | Schottky junction | 600 | 0.1 | - | 23 s/28 s | [12] |
| MoS_2_/WS_2_ | p–n heterojunction | 532 | 4.36 | $4.36\times{10}^{13}$ | 4 ms | [13] |
| WSe_2_ | homojunction | 532 | 11 | $4.4\times{10}^{10}$ | 0.18 ms/9.22 ms | [14] |
| PdSe_2_ | Schottky junction | 532-1470 | 52.4 | $5.17\times{10}^{11}$ | 0.24 ms/0.72 ms | This work |

**Reference**

1. Oyedele, A.D., et al., PdSe_2_: pentagonal two-dimensional layers with high air stability for electronics. Journal of the American Chemical Society, 2017. **139**(40): p. 14090-14097.

2. Kim, H.-g. and H.J. Choi, Quasiparticle band structures of bulk and few-layer PdSe_2_ from first-principles G W calculations. Physical Review B, 2021. **103**(16): p. 165419.

3. Wang, H., et al., Junction Field‐Effect Transistors Based on PdSe_2_/MoS_2_ Heterostructures for Photodetectors Showing High Responsivity and Detectivity. Advanced Functional Materials, 2021. **31**(49): p. 2106105.

4. Huang, Y., et al., Effects of organic molecules with different structures and absorption bandwidth on modulating photoresponse of MoS_2_ photodetector. ACS applied materials & interfaces, 2016. **8**(35): p. 23362-23370.

5. Dai, M., et al., Two-Dimensional van der Waals Materials with aligned in-plane polarization and large piezoelectric effect for self-powered piezoelectric sensors. Nano letters, 2019. **19**(8): p. 5410-5416.

6. Zhao, S., et al., Highly Polarized and Fast Photoresponse of Black Phosphorus‐InSe Vertical p–n Heterojunctions. Advanced Functional Materials, 2018. **28**(34): p. 1802011.

7. Wang, Y., et al., A room-temperature near-infrared photodetector based on a MoS_2_/CdTe p–n heterojunction with a broadband response up to 1700 nm. Journal of Materials Chemistry C, 2018. **6**(18): p. 4861-4865.

8. Mahyavanshi, R.D., et al., Photovoltaic Action With Broadband Photoresponsivity in Germanium-MoS_2_ Ultrathin Heterojunction. IEEE Transactions on Electron Devices, 2018. **65**(10): p. 4434-4440.

9. Zhong, J., et al., High-performance polarization-sensitive photodetector based on a few-layered PdSe_2_ nanosheet. Nano Research, 2020. **13**(6): p. 1780-1786.

10. Lee, Y., et al., Trap-induced photoresponse of solution-synthesized MoS_2_. Nanoscale, 2016. **8**(17): p. 9193-9200.

11. Zeng, L.H., et al., Controlled synthesis of 2D palladium diselenide for sensitive photodetector applications. Advanced Functional Materials, 2019. **29**(1): p. 1806878.

12. Kang, Z., et al., MoS_2_-based photodetectors powered by asymmetric contact structure with large work function difference. Nano-micro letters, 2019. **11**(1): p. 1-12.

13. Wu, W., et al., Self-powered photovoltaic photodetector established on lateral monolayer MoS_2_-WS_2_ heterostructures. Nano Energy, 2018. **51**: p. 45-53.

14. Tan, C., et al., A self-powered photovoltaic photodetector based on a lateral WSe_2_-WSe_2_ homojunction. ACS Applied Materials & Interfaces, 2020. **12**(40): p. 44934-44942.
